# Notch signaling triggered via the ligand DLL4 impedes M2 macrophage differentiation and promotes their apoptosis

**DOI:** 10.1186/s12964-017-0214-x

**Published:** 2018-01-10

**Authors:** Sylvain Pagie, Nathalie Gérard, Béatrice Charreau

**Affiliations:** 1 Centre de Recherche en Transplantation et Immunologie UMR1064, INSERM, Université de Nantes, 30 bd J. Monnet, 44093 Nantes, France; 2 Institut de Transplantation Urologie Néphrologie (ITUN), CHU Nantes, Nantes, France

**Keywords:** Notch pathway, Notch1, DLL4, Il-4, M2 macrophage, Macrophage, Polarization, JAK/stat, Apoptosis

## Abstract

**Background:**

Notch signaling controls many cellular processes, including cell fate determination, cell differentiation, proliferation and apoptosis. In mammals, four Notch receptors (Notch 1–4) can interact with five distinct ligands [Jagged1, Jagged2, Delta-like 1 (DLL1), DLL3, and DLL4]. We previously reported that Notch activation is modulated in endothelial cells and monocytes during inflammation and showed that inflammation upregulates DLL4 on endothelial cells. DLL4 promotes differentiation of blood monocytes into proinflammatory M1 macrophages. Here, we further investigated the ability of DLL4 to interfere with the polarization of blood monocytes into immunosuppressive M2 macrophages.

**Methods:**

Human blood monocytes were differentiated in vitro into M0 macrophages and then polarized into M1 or M2 macrophages with LPS/IFNγ and IL-4, respectively. Polarization steps were performed in the presence of immobilized recombinant DLL4. Immune phenotype and apoptosis of macrophage subsets were analyzed and quantified by flow cytometry. Regulatory effects of DLL4 on gene expression, cell signaling and apoptotic pathways were investigated by QPCR and western blots.

**Results:**

The phenotype of M2 macrophages was subject to specific alterations in the presence of recombinant DLL4. DLL4 inhibits the upregulation of IL-4 induced M2 markers such as CD11b, CD206, and CD200R. Survival of macrophages upon M2 polarization was also strongly reduced in the presence of DLL4. DLL4 induces a caspase3/7-dependent apoptosis during M2 but not M1 macrophage polarization. The Notch ligand DLL1 has no apoptotic effect. Both DLL4 signaling via Notch1 as well as DLL4-mediated apoptosis are Notch-dependent. Fully differentiated M2 macrophages became resistant to DLL4 action. Mechanistically, DLL4 selectively upregulates gene expression in macrophages upon M2 polarization, thereby affecting the Notch pattern (Notch1, 3, Jag1), activity (HES1), and transcription (IRF5, STAT1). The pro-apoptotic effectors Bax and Bak and the BH3-only proteins Bid and Bim seem to convey DLL4 apoptotic signal.

**Conclusion:**

Interplay between the DLL4/Notch and IL-4/IL-4R signaling pathways impairs M2 differentiation. Thus, DLL4 may drive a Notch-dependent selection process not only by promoting M1 macrophage differentiation but also by preventing M2 macrophage differentiation through inhibition of M2-specific gene expression and apoptotic cell death.

## Background

Macrophages are key players of innate immunity and play a central role in inflammation and host defense against pathogens. Macrophages also contribute to tissue homeostasis by participating in tissue repair and by regulating metabolic functions. An important feature of the monocyte/macrophage lineage is their diversity and plasticity. These varying functional phenotypes may be acquired through specific environmental cues [1–3]. Mirroring the Th1/Th2 classification of T cells, macrophages may undergo either classical (M1) or alternate (M2) activation. Beside this classification, macrophages can adopt a spectrum of activation states ranging from M1 to various alternatively activated M2 macrophages depending on the nature of stimuli in the environment [1]. M1 macrophages express high level of proinflammatory cytokines and reactive oxygen species. M1 macrophages promote Th1 responses and have an important role in combating bacteria and tumors. In contrast, M2 macrophages display immunoregulatory properties and are involved in parasite control, tissue remodeling and tumor progression. Changes in the balance of macrophage activation are associated with several pathological conditions including cancer, infection and autoimmunity [4]. M1 macrophages are involved in the initiation and persistence of inflammatory disorders while M2 macrophages are associated with the resolution or chronicity of low-grade inflammation but also with tumor progression. The molecular basis of macrophage polarization is complex and includes transcription factors, signaling pathways, and epigenetic as well as post-transcriptional regulation. At the molecular level, a balance between the Signal Transducer and Activator of Transcription (STAT) proteins STAT1 and STAT3/6 regulates M1 versus M2 polarization defining their inflammatory and immunosuppressive functions, respectively. The nuclear factor-κB (NF-κB) and STAT1 are involved into M1 activation into cytotoxic and inflammatory cells. In contrast, the immunosuppressive and pro-tumor activities of M2 result from STAT3 and STAT6 activation. Krüppel-like factor (KLF) 4 cooperates with STAT to induce M2 and inhibit M1 genes, respectively. KLF2 also inhibit NF-κB activity. Macrophage differentiation is a dynamic process and macrophages can switch from one phenotype to another according to requirements [5, 6].

Notch signaling is also implicated in macrophage polarization. Notch has an essential role in specifying cell fate at multiple stages in a broad array of cell types, [7] including immune cells [8, 9]. The mammalian family of Notch proteins includes four receptors (Notch1–4) and a set of ligands comprising of Jagged (Jag1 and 2) and Delta-like members (DLL1, 3, and 4). Notch receptors and ligands are transmembrane proteins with extracellular domains required for juxtacrine ligand-receptor interactions. This cell-to-cell interaction induces a sequence of cleavages of the receptor mediated by proteases such as γ-secretase, which results in the release of the Notch intracellular domain (NICD) into the nucleus. In canonical Notch pathway activation, nuclear NICD binds the RBP-J DNA-binding protein (also known as CSL or CBF1) to form a transcriptional activator through the recruitment of coactivator proteins [10]. Alternative mechanisms of Notch signaling (i.e. non canonical), RBP-J–independent, have been described [10]. Forced activation of Notch signaling increased M1 macrophages, while macrophages deficient in canonical Notch signaling showed M2 phenotypes [11]. Macrophage expression of Notch receptors (such as Notch1) and ligands, and the activation of canonical Notch signaling contribute to M1 gene expression and cytokine production [12–14]. NICD also interacts with HIF-1α and increases glycolytic activity involved in M1 activation [15]. Notch activation can be initiated in macrophages by toll like receptor (TLR) activation [14, 16]. Nevertheless, whether the Notch pathway plays a regulatory role in M2 macrophage polarization remains mostly unknown.

DLL4 is the only Notch ligand with a restricted pattern of cellular expression and is mostly expressed by endothelial and myeloid cells [17, 18]. DLL4 also exhibits a specific and high affinity to Notch compared to Jag1 and DLL1 and is involved in endothelial/macrophage interactions during angiogenesis [19–21]**.** Our previous studies highlighted the interplay between Notch signaling and inflammation in endothelial cells and in monocytes/macrophages. We reported that Notch activation in monocytes, which is triggered via endothelial DLL4 in conjunction with IL-6, promotes the proinflammatory M1 macrophage genotype and phenotype [22]. Our data also supported a Notch-dependent crosstalk between activated endothelium and intravascular macrophages during microvascular inflammation in cardiac allografts. The present study focused further on the contribution of DLL4 to macrophage differentiation by analyzing the ability of DLL4 to interfere with M2 polarization. Overall our findings suggest that DLL4 may drive a Notch-dependent selection process not only by promoting macrophage differentiation towards M1 but also by preventing M2 differentiation. Two mechanisms appear to operate to preclude M2 polarization: inhibition of M2-specific gene expression and apoptotic cell death.

## Methods

### Reagents and antibodies

Tissue culture plates were coated for 4 h with recombinant human DLL4 (rhDLL4) and DLL1 (rhDLL1) proteins purchased from R&D Systems (R&D Systems Europe Ltd., Abingdon, UK), diluted into PBS (5 μg/mL). Pharmacological inhibitors of γ-secretase (N-[N-(3,5-Difluorophenacetyl)-L-alanyl]-Sphenylglycine t butyl ester; DAPT, Sigma Aldrich, Lyon, France) and of JAK1/JAK2 (Ruxolitinib, Invivogen, Toulouse, France) were used at 0.5 μg/mL and 0.1 μg/mL, respectively. For the inhibition of γ-secretase, macrophages were incubated with DAPT (0.5 μg/mL; Sigma Aldrich) for the 48 h during or for 24 h following M1 or M2 polarization. Similarly, inhibition of JAK/STAT signaling pathway was achieved by adding Ruxolitinib (0.1 μg/mL; Invivogen) during the polarization step. Macrophage Colony Stimulating Factor (M-CSF), IL-4 and IL-10 were from R&D Systems.

### Purification of human monocytes, differentiation and polarization protocols

Monocytes (CD14^+^) were issued from healthy blood donors at the Etablissement Français du Sang (EFS-Pays de la Loire, Nantes, France). PBMC fraction was isolated by standard Ficoll isolation technique and then CD14^+^ monocytes were purified by counterflow centrifugal elutriation with a minimal purity of ≥87% (DTC-core-facility, CIC BT0503, Nantes, France). For differentiation, purified monocytes were grown in RPMI1640 medium supplemented with 20% Fetal Bovine Serum, 100 U/mL Glutamine, 100 μg/mL penicillin-streptomycin, 1X non-essential amino acids (Life Technologies, Cergy-Pontoise, France), 1 mM sodium pyruvate (Life Technologies) and M-CSF (100 ng/mL, R&D Systems) for 7 days on 9 cm^2^ dishes (1.10^6^ cells/cm^2^) to differentiate into M0 macrophages. For M1/M2 polarization, M0 macrophages (2.5 × 10^5^ cells/cm^2^) were then cultured for an additional 48 h in RPMI supplemented with 10% FBS and IFN-γ (100 U/mL; Imukin, Boehinger, Ingelheim, Germany) plus LPS (100 ng/mL; Sigma Aldrich) for M1 differentiation or with either IL-4 (20 ng/mL; R&D Systems) or IL-10 (20 ng/mL; R&D Systems) for M2a and M2c differentiation, respectively.

### Messenger RNA and quantitative RT-PCR

Total RNAs were isolated using Trizol reagent (Life Technologies), phenol-chloroform extraction and isopropanol precipitation. RNAs were analyzed using a microchip capillary electrophoresis system (Caliper LabChip GX, PerkinElmer, Waltham, MA, USA). RNA with RQS >7 (RNA Quality Score) were treated with ribonuclease-free Turbo-DNase (Life Technologies) before reverse transcription (RT). RTs were performed with Moloney murine leukemia virus reverse transcriptase (Life Technologies) according to the manufacturer’s instructions. Real-time quantitative PCR was performed with a ViiA™ 7 sequence detection application program using the following labeled ready-to-use primers/probe mixes (Life Technologies): Notch1 (Hs01062014_m1), Notch2 (Hs01050702_m1), Notch3 (Hs01128541_m1), Notch4 (Hs00270200_m1), DLL1 (Hs00194509_m1), DLL3 (Hs00213561_m1), DLL4 (Hs00184092_m1), Jag1 (Hs01070036_m1), Jag2 (Hs00171432_m1), HES1 (Hs00172878_m1), HEY1 (Hs01114113_m1), IRF5 (Hs00158114_m1), STAT1 (Hs01013996_m1), SOCS3 (Hs02330328_s1), TNF (Hs01113624_g1), IL-6 (Hs00174131_m1), IL-1β (Hs0155410_m1), CD40 (Hs00334176_m1), c-myc (Hs00811069_g1). QPCR for β actin (Actb) (Hs99999903_m1) was used as an endogenous control and for data normalization. Relative gene expression was calculated according to the 2^−ΔΔCt^ method.

### Phenotype and apoptosis analysis by flow cytometry

For direct immunofluorescence labeling, macrophages were stained with the following fluorescence-labeled anti-human mAbs: anti-CD40-FITC, anti-CD64-A700, anti-CD80-V450 and anti-CD86-APC, anti-CD11b-pacific blue, anti-CD163-PE, anti-CD206-FITC (all from BD Biosciences, San Diego, CA) and anti-CD200R-APC Abs (Serotec, Oxford, UK). For Notch1 receptor analysis, macrophages were immunostained using rabbit anti-Notch1 (1:100 dilution, ab52627 from Abcam, Cambridge, UK) Abs and Alexa-488 labeled anti-rabbit IgG as secondary antibodies. For apoptosis analysis, cells were incubated with Annexin V/propidium iodide following the manufacturer’s recommendations (Life Technologies). Cells stained with, isotype-matched, irrelevant Abs were used as negative controls. Fluorescence was measured by flow cytometry after gating on FSC/SSC parameters using a FACS LSR II® (BD Biosciences) and next analyzed using FlowJo® VX software (Tree Star, Inc., Ashland, OR, USA).

### Caspase 3/7 activity

For time-lapse experiments, cells (2.5 × 10^5^), pre-incubated with or without DAPT, were plated on 8-wells Ibidi μ-Slides (Ibidi, Biovalley Nanterre, France) pre-coated with rhDLL4, rhDLL1 or PBS. One drop of DEVD substrate (CellEvent®Caspase-3/7 Green, Molecular probes, Life technologies) was added to the culture medium supplemented or not with IL-4. Intracellular Caspase 3/7 activity detected by nuclear fluorescence was recorded in live cells at 37 °C for 48 h. Cells were illuminated every 10 min using a xenon lamp a 480/520 nm excitation filter. Emission at 525/530 nm was recorded for analysis of caspase 3/7 activity and captured with a Cool Snap HQ2 camera (Roper, Tucson, AZ, USA). Images were analyzed using Metamorph 7.5.6© software (Molecular Devices, Sunnyvale, CA, USA).

### Western blotting

After treatments, cells were lysed in RIPA buffer containing protease and phosphatase inhibitors (PIC cocktail, Sigma-Aldrich). Protein concentration was determined using BCA™ protein assay reagent (Pierce, Thermo Fisher Scientific). Cell lysates were resolved by SDS-PAGE (10%), and proteins were transferred to nitrocellulose membranes (ECL Hybond; Amersham, Little Chalfont, UK) using a Trans-Blot SD semi-dry electrophoretic transfer cell (Bio-Rad, Marne-la-Coquette, France). Then, membranes were subjected to immunoblot analysis using the following primary antibodies at dilution 1:1000: anti-Notch1 (C44H11), anti-HES1, anti-Bak, anti-Bax, anti-Bid, anti-Bim, anti-phospho-Bad, anti-Puma, anti-c-myc from Cell Signaling Technology (Leiden, NL) and anti-β-actin and anti-GAPDH (sc-32,233) from Santa Cruz Biotechnology (Dallas, TX, USA). Revelation was performed using appropriate peroxidase-conjugated secondary antibodies. Antibody-bound proteins were detected and analyzed using an enhanced chemiluminescence (ECL) kit (Amersham) and luminescent image analyzer LAS-4000 (Fujifilm, Tokyo, Japan).

### Statistical analysis

All experiments were representative of at least 3 independent experiments**.** Data are expressed as mean ± SD and compared using non-parametric Mann-Whitney test and Kruskal-Wallis test (with Dunn’s multiple comparison post-test) when multiple conditions were compared. A *p* value <0.05 was considered statistically significantly different. In figures (*) denotes *p* < 0.05, (**) denotes *p* < 0.01, (***) denotes *p* < 0.005.

## Results

### DLL4 impairs IL-4-mediated differentiation into M2 macrophage

**Fig. 1:**
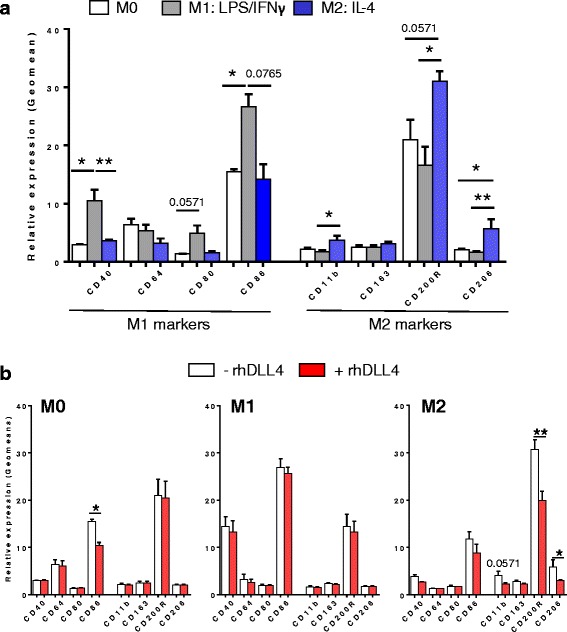
DLL4 impairs M2 macrophage polarization. **a** Phenotype analysis of macrophages before (M0) and after polarization in M1 or M2 macrophages for 24 h with LPS and IFN-γ or IL-4, respectively (*n* = 5). **b** Phenotype analysis of M0, M1 and M2 macrophages polarized during 24 h with (+rhDLL4) or without (−rhDLL4) coated rhDLL4 (*n* = 5). Data are expressed as fold increases ± SD from 5 independant experiments. M1-specific and M2-specific makers are indicated. Mann-Whitney test was used for statistical analysis of the results (*p < 0.05 and ** if *p* < 0.01)

To investigate the interplay between Notch and macrophage phenotype, monocytes were purified from blood samples issued from healthy volunteers (EFS des Pays de la Loire, Nantes, France). Monocytes were sequentially differentiated into M0 macrophages using M-CSF for 7 days and then polarized into M1 macrophages with IFNγ and LPS or into M2 macrophages (M2a subtype) using IL-4 for 48 h, as previously described [23]. Figure 1a shows the respective phenotype of M1 and M2 macrophages at 24 h post-differentiation compared to M0 harvested at the end of M-CSF treatment. Four M1-specific markers were investigated: CD40, CD64, CD80 and CD86. In our conditions, polarized M1 macrophages displayed changes at phenotypic level compared to M0 that includes a significantly enhanced expression for CD40 and CD86 and a trend of increased CD80 (*p* = 0.0571). In contrast, LPS/IFNγ induced no change in M2 markers. Conversely, polarization in the presence of IL-4 increased three out of the four M2-specific markers tested (CD11b, CD200R and CD206). IL-4 induced no change in the M1 markers. Thus, our protocol allows for macrophages to terminally differentiate into M1 that display CD40^high^,CD64^high^, CD80^high^ CD86^high^ phenotype and into M2a with a CD11b^high^, CD206^high^, CD200R^high^ phenotype.

Ectopic expression of ligands for Notch may lead to lateral activation (cis-activation) of Notch in the transfected cells [24]. To exclude this, the effect of DLL4 was investigated by performing macrophage differentiation with either LPS/IFNγ or IL-4 in the presence of recombinant human DLL4-Fc (rhDLL4) immobilized on culture plates to mimic cell-bound DLL4. As a result, we found that DLL4 significantly precludes the upregulation of CD11b, CD206 and CD200R expression in response to IL-4 that all remained near the baseline observed on M0 (Fig. 1b). DLL4 did not impair the basal expression of CD11b, CD206 and CD200R on M0 or M1 macrophages. Moreover, when M1 polarization was performed in the presence of rhDLL4 no significant change was found in M1-specific markers (CD40, CD64, CD80, CD86). These findings suggest an inhibitory role for DLL4 and Notch signaling on the IL-4 signaling pathway*.* This data unveils that Notch signaling through DLL4 is a regulatory mechanism of macrophage differentiation that play an important role in preventing M2 polarization.

### DLL4 selectively triggers apoptosis of M2 macrophages.

**Fig. 2:**
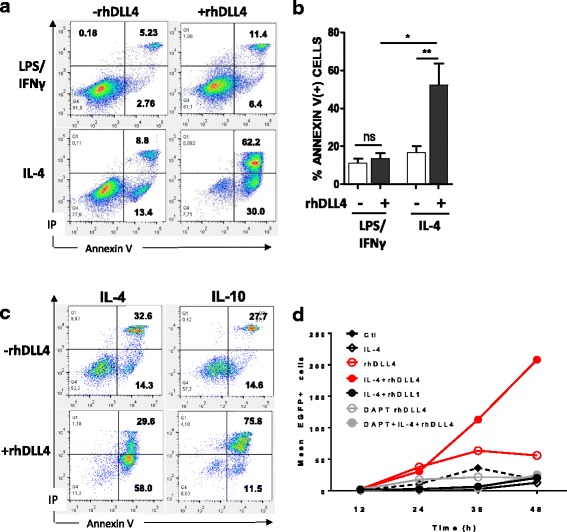
DLL4 triggers specific apoptosis during M2 macrophage polarization. Analysis of apoptosis after M1 (LPS/IFNγ) or M2 (IL-4) polarization with (+rhDLL4) or without (−rhDLL4) coated rhDLL4. **a**, **b**, **c** After 48 h of polarization cells were stained by Annexin V/Propidium Iodide (IP) and the fluorescence was measured by flow cytometry. **a** Results shown are representative dot plots from one out of 5 independent experiments. **b** Percentage of Annexin V positive cells among polarized M1 or M2 subsets (means ± SD; *n* = 5). **c** Analysis of apoptosis in M2 macrophages polarized with IL-4 or IL-10 with or without coated rhDLL4 (*n* = 3). Mann-Whitney test was used for statistical analysis of the results (*p < 0.05 and ** if *p* < 0.01). **d** Time lapse analysis of caspase 3/7 activity upon M2 polarization. Cells were polarized for 48 h into M2 macrophages (IL-4) or maintain at M0 stage (-IL-4) with or w/o immobilized rhDLL4 or rhDLL1 and with or w/o γ-secretase inhibitor (DAPT). Curves represent the number of apoptotic cells in each condition during the time of the experiment. Data are expressed as means ± SD of EGFP+ cells (activated caspase 3 positive cells) from at least 3 fields per wells

Next, we sought to define whether DLL4 could also affect macrophage survival and promote apoptosis. To this aim, apoptosis was measured using annexin-V immunostaining and by the kinetic analysis of caspase-3/7 activity in live cells by time-lapse video-microscopy. Incubation with rhDLL4 during IL-4-mediated M2 differentiation triggers a dramatic apoptosis of M2 cells leading to up to 30% early apoptosis (IP^−^/AnnexinV^+^) and 62.2% of late apoptosis/necrosis (IP^+^/AnnexinV^+^) in cells compared to those without rhDLL4 (Fig. 2a and b). In parallel experiments, macrophages incubated with rhDLL4 during the polarization into M1 cells also exhibited slight changes in cell viability. Although moderate in magnitude (early apoptosis, from 2.76% to 6.4%; late apoptosis, from 5.23% to 11.4%), DLL4 may promote the apoptosis of M1. Interestingly, when IL-10 is used instead of IL-4 to induce M2c instead of M2a differentiation, DLL4 drives apoptosis differently (Fig. 2c). DLL4 mainly drives the early apoptosis of IL-4 induced M2, while driving the late apoptosis of IL-10 induced M2. This may indicates different Notch-related apoptosis mechanisms in the two types of M2 macrophages supporting the idea that DLL4/Notch signal selectively impairs anti-apoptotic/pro-survival signals mediated by IL-4 or IL-10 in differentiating macrophages.

Time course analysis of caspase-3/7 activity by videomicroscopy in live cells revealed that cell apoptosis started rapidly after incubation with IL-4 in the presence of DLL4 and reached a maximal level at 48 h. We used this dynamic analysis to test whether the apoptotic effect was specific to DLL4 or could be achieved using another Notch ligand. To this aim, experiments were repeated using recombinant DLL1 instead of DLL4. As shown in the Fig. 2d, in contrast to DLL4, DLL1 had no effect on cell apoptosis. Moreover, in the presence of DAPT, an inhibitor of γ-secretase, DLL4-mediated M2 apoptosis was abrogated indicating that DLL4 triggers M2 apoptosis via Notch signaling. Therefore, the regulatory control of M2 differentiation seems to be DLL4-specific and Notch-dependent and results in a blockade in M2-specific molecules and an induction of apoptosis.

### Interplay between DLL4/Notch and IL-4/IL-4R signals at the JAK/STAT level during the M2 differentiation process

**Fig. 3:**
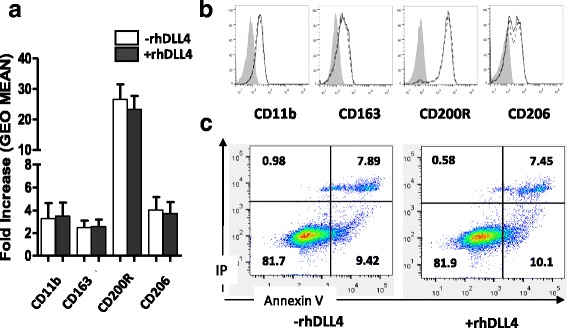
Fully differentiated M2 macrophages resist to DLL4-induced apoptosis. Macrophages were polarized into M2 macrophages with IL-4 for 48 h then cultivated for 24 h with (+rhDLL4) or without (−rhDLL4) immobilized rhDLL4. **a** Quantitative analysis from flow cytometry of four M2 markers (CD11b, CD163, CD200R, CD206) expressed at the surface of M2 macrophages (mean ± SD; *n* = 3). Results are expressed as the fold increase from 3 experiments **b** Representative histograms of the M2 markers expression on M2 macrophages cultivated for 72 h with IL-4 and without rhDLL4 (Black line) or 48 h plus 24 h more on immobilized rhDLL4 (Dashed line). **c** Apoptosis M2 macrophages. At 72 h, M2 macrophages were stained with Annexin V/Propidium Iodide (IP) and the fluorescence was measured by flow cytometry. Results are expressed as dot plots representative of 3 independent experiments

**Fig. 4:**
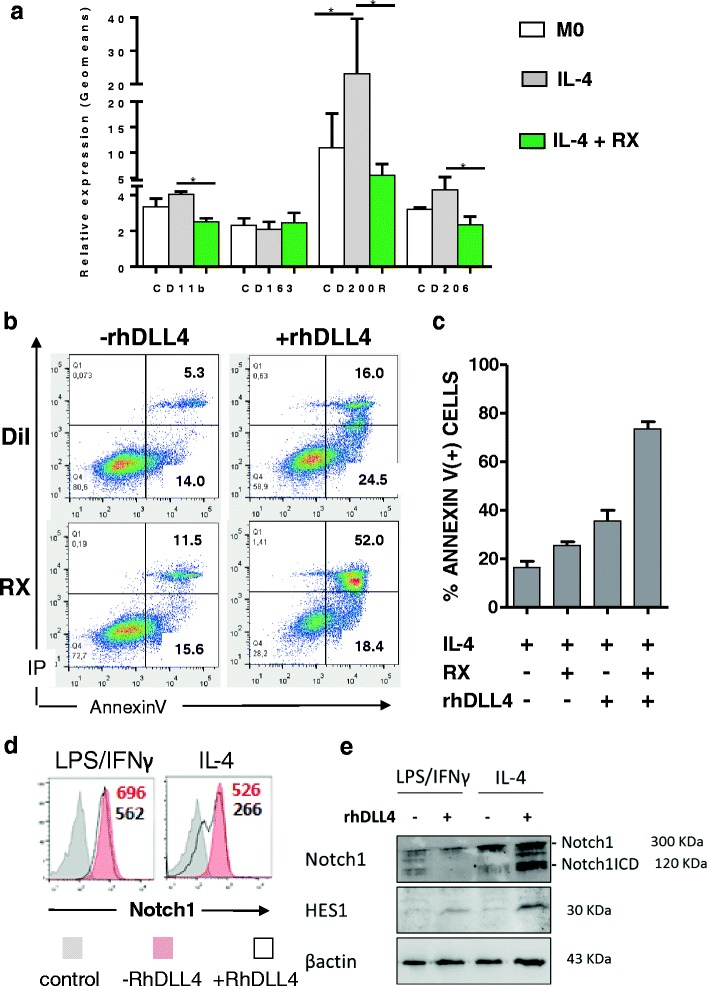
Interplay between JAK/STAT and DLL4/Notch1 signaling. **a**, **b**, **c** Inhibition of JAK/STAT signaling pathway potentiates DLL4-mediated M2 apoptosis. Cells were polarized into M2 macrophages by IL-4 with or without the JAK/STAT inhibitor Ruxolitinib (RX) and with (+rhDLL4) or without (−rhDLL4) coated rhDLL4. **a** Expression of M2 markers at the membrane of M0 or M2 polarized macrophages with or without Ruxolitinib. Results from flow cytometry show geometric means of fluorescence expressed as relative expression (mean ± SD, *n* = 3). **b** Apoptosis of M2 polarized cells upon inhibition of JAK/STAT with Ruxonitilb (RX). After 24 h of polarization cells were stained with Annexin V/Propidium Iodide (IP). Results are representative dot plots from 3 independent experiments. **c** Histogram representing percentage of Annexin V positive cells (*n* = 3). **d** Flow cytometry of Notch1 surface expression on M1 and M2 differentiated for 48 h in the presence of immobilized DLL4. **e** Representative western blots for Notch1, N1ICD, HES1 and β actin in M1 and M2 Results are representative data from 3 independent experiments

Next, we tested whether DLL4 may still convey its action on fully differentiated M2 macrophages. Thus IL-4-induced M2 macrophages were incubated with DLL4 and the impact of DLL4 signaling on both M2 phenotype and apoptosis was measured. The expression of M2-specific markers (CD11b, CD163, CD200R and CD206) was unchanged in the presence of DLL4 indicating that DLL4 induces no change in a well established M2 phenotype (Fig. 3a and b). Similarly, DLL4 was not able to induce apoptosis of fully differentiated M2 macrophages (Fig. 3c). This suggests that differentiated M2 macrophages became resistant to DLL4 action. These findings support the hypothesis that DLL4/Notch signaling interferes with the IL-4/IL-4R signaling during the differentiation process. Supportive of this hypothesis, we further observed that inhibition of the JAK/STAT pathway, a downstream signaling event common to IL-4 and IL-10 and to many pro-survival signals, significantly potentiating the inhibitory effects of DLL4 on the polarization of M2 macrophages (Fig. 4). Ruxolitinib a pharmacological inhibitor of JAK1/JAK2 efficiently prevents IL-4 signaling conducting M2 differentiation as attested by a potent inhibition of CD200R and CD206 expression level. Thus inhibition of JAK1/JAK2 recapitulates the effect of DLL4 on CD200R and CD206. Ruxolitinib alone only slightly enhances macrophage apoptosis. Ruxolitinib strongly increases the percentage of apoptotic cells mediated by DLL4 in the presence of IL-4 (72% versus 38%). These data provides initial evidence for an interaction between DLL4/Notch and IL-4/IL-4R signals at the JAK/STAT level.

### DLL4/Notch1 axis influences Notch profile and regulates signaling mediators upon M2 differentiation

**Fig. 5:**
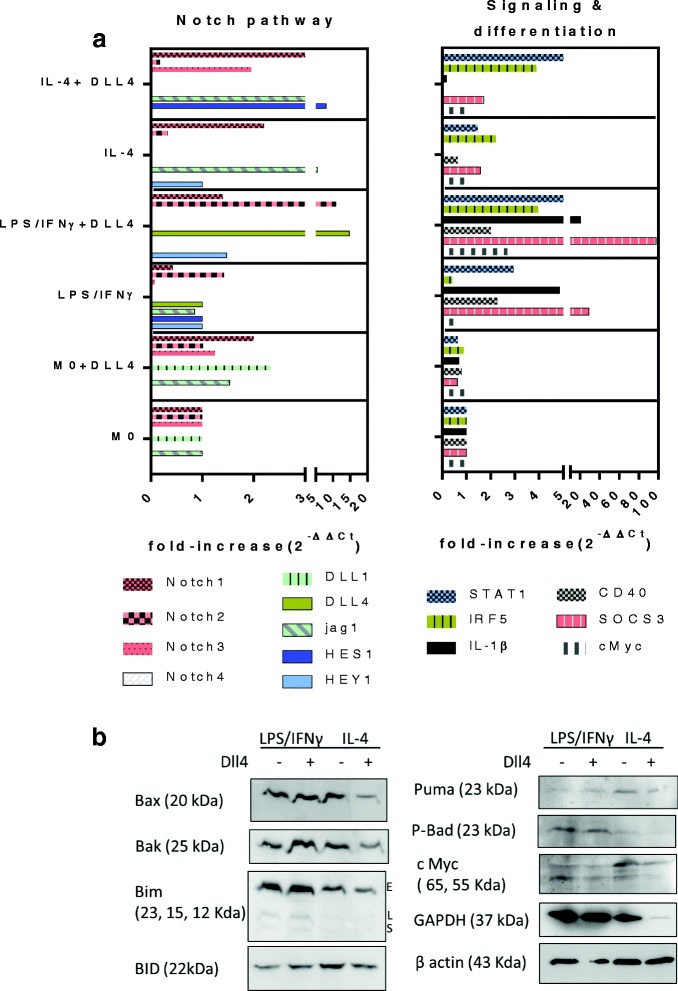
Regulatory network mediated by DLL4 in macrophage subsets. **a** QRTPCR analyses for Notch family members and signaling molecules. Macrophages (M0) were incubated with LPS + IFNγ (M1), IL-4 (M2) and with (+) or without (−) DLL4 for 24 h. Results shown are the means of triplicate experiments. The data were normalized using a housekeeping gene (β actin) and expressed as 2^-ΔΔCt^ using untreated M0 as reference. **b** Representative western blots for pro-apoptotic and effector molecules in M1 and M2 after polarization with LPS + IFNγ or IL-4 for 48 h and with or without immobilized DLL4

Gene expression analysis was performed by quantitative RT-PCR to identify the regulations mediated through DLL4/Notch in macrophages. To this aim, macrophages before (M0) and after polarization with LPS/IFNγ (M1) or IL-4 (M2) with or without rhDLL4 were analyzed. A set of 15 transcripts encoding Notch receptors, ligands and effectors and signaling molecules were quantified (Fig. 5a). We found that DLL4 selectively influences RNA transcription during M1 versus M2 differentiation. Upon M1 polarization, DLL4 increased mRNAs for DLL4, Notch2 and HEY1 consistent with our previous studies [25, 22]. Upon M2 polarization DLL4 increased mRNAs for Notch1, 3 and HES1. Therefore, DLL4 seems to differentially activate the Notch pathway on macrophages subsets. QPCR also indicate that DLL4 potentiates the upregulation of transcripts associated with inflammation (STAT1, IRF5, IL-1β and SOCS3) in M1 and to a lesser extent (STAT1, IRF5) in M2.

Since DLL4 upregulates Notch1 mRNA during polarization, we speculated that DLL4 might signal through Notch1 in these cells. To test this hypothesis, we analyzed the surface expression of Notch1 during the differentiation in the presence of DLL4. Incubation with recombinant DLL4 during the differentiation process results in a strong decrease in Notch1 membrane expression on M2 macrophages while only a modest decrease was found in M1 macrophages (Fig. 4d). A decrease in Notch1 receptor at the cell surface may reflect receptor cleavage following ligand/receptor interaction and its subsequent activation of Notch signaling. Using antibodies directed against the intracellular domain of Notch1 (N1ICD) in Western blotting assays, we showed that DLL4 promotes the release of N1ICD in response to IL-4 but not in response to LPS/IFNγ (Fig. 4e). Upregulation of HES1 confirmed Notch activation via DLL4. These data suggest that DLL4 mediates Notch signaling in macrophages via Notch1 as previously reported in other cell types [26]. Apoptotic pathways was further explored by western blots performed on cytosolic extracts from M1 and M2 polarized subsets (Fig. 5b). During M2 polarization, DLL4 drives a significant drop in cytosolic levels of proapoptotic effector proteins (Bax and Bak) and BH3-only proteins (Bim, Bid). This drop is consistent with the translocation of these proteins during the apoptosis cascade. Interestingly, parallel to bcl2 family members, a strong decrease in GAPDH was also observed. Nuclear translocation of GAPDH suggests a possible contribution of GAPDH to the apoptotic process.

## Discussion

Previous studies exploring the role of the Notch pathway in immunity have been mainly focused on lymphocytes [9]. Notch signaling can also influence myeloid cell differentiation and its contribution to the specification of macrophage subtypes and functions is emerging [11, 27, 28]. In a seminal study, Fung et al. reported on the expression of multiple Notch receptors and ligands on human macrophages [27]. They showed a marked increase in DLL4 expression on human primary macrophages in response to proinflammatory stimuli (LPS, IL-1β, and Low Density Lipoproteins). This study also characterized DLL4 as a marker of M1-type macrophages and showed the detection of DLL4 in human atherosclerotic plaques suggesting possible homotypic and heterotypic roles for DLL4 in activated macrophages [27]. Consistent with these data, we recently reported increased DLL4 expression in both endothelial cells and macrophages during microvascular inflammation suggesting that DLL4 may be an activation marker and could play a role in inflammatory endothelial/macrophage interactions [22]. Forced activation of Notch signaling increased IL-12 producing M1 macrophages, no matter whether M1 or M2 stimuli were applied. When Notch signaling was blocked, the M1 inducers induced M2 response at the expense of M1 response [11]. Here we show that the Notch ligand DLL4 is a potent inhibitor of macrophage differentiation into M2-type macrophages. This repressive effect seems to result from both an inhibition of M2 gene expression and a selective induction of cell apoptosis.

As a first result, our study indicated that induction of M2 polarization by IL-4 in the presence of immobilized recombinant DLL4 leads to a significant inhibition of M2 phenotype. DLL4 selectively prevents the upregulation of CD11b, CD200R and CD206 promoted by IL-4. In contrast, DLL4 has no effect on M1 markers regulated by LPS and IFNγ. Likewise, the phenotype of M0 was found unchanged in the presence of DLL4 except a slight decrease for CD86. Our findings are thus consistent with previous data demonstrating in T cells that DLL4 signals drive Th17 differentiation through the upregulation of RORγt while at the same time limiting Th2 cytokine production [29]. Together these data substantiate evidence for DLL4 in orienting both innate and adaptive immune cells toward a proinflammatory phenotype also through a negative regulation of immunosuppressive cells and cytokines.

Furthermore, associated with the blockade of M2 phenotype, DLL4 induces the cell death of the vast majority of macrophages differentiating in the presence of IL-4 (up to 92% of total apoptosis in our conditions, Fig. 2). Apoptosis mediated by DLL4 is caspase-dependent and Notch-dependent. Apoptosis was observed with DLL4 but not with DLL1. Central in tissue homeostasis, Notch is essential to maintain a sensitive balance between cellular proliferation, survival and apoptosis. Consequently, Notch activation has been shown to promote or inhibit apoptosis depending on cell type and the surrounding environment [7, 25, 30, 31]. For instance in T cells, Helbig and colleagues have shown that Notch controls the size of the CD4^+^ T-cell population, predominantly by protecting already expanded clones from apoptosis. This anti-apoptotic effect is efficiently mediated by recombinant DLL4 and to a lesser extent by DLL1 [32]. Notch also promotes expansion of CD8^+^ T cells, in part, by protecting these cells from cell death supporting the idea that Notch activation via DLL4-Fc has a general role in controlling the viability of activated T cells. An important point is the nature of the Notch receptor that conveys the DLL4 apoptotic signal in lL-4 induced M2 cells. According to the literature, Notch1 and Notch4 are the major ligands for DLL4 [33]. Overall, our experiments using QPCR, flow cytometry and Western blots mostly support the implication of a DLL4/Notch1 axis in the apoptosis of IL-4-treated macrophages with a possible involvement of HES1 as effector. Interestingly, interplay between DLL4 and Notch1 has been recently described in migrating epithelium where leader cells express high levels of DLL4 [26]. In this model, DLL4/Notch1 signaling provides an autoregulatory mechanism to maintain the density of leader cells during collective migration. If the density of DLL4-expressing leader cells is high, the Notch1 level in neighboring cells will be enhanced, in turn inhibiting DLL4 expression. Whether similar autoregulation may occur in the interplay between M1/M2 macrophages remains to be explored.

Accumulating evidence supports the existence of important but incompletely understood crosstalk between Notch and other signaling pathways such as MAPK, PI3K/Akt, and NF-κB [34]. Our findings provide evidence for a crosstalk between IL-4/IL-4R signaling and the Notch pathway mediated by DLL4. IL-4 signaling is well known and is initiated after receptor oligomerization, which involves JAK1 and JAK2 [35]. This can be inhibited by SOCS-1. The induction of genes by IL-4 involves activation of STAT proteins. JAK1 regulates STAT3 and STAT6 activation, by tyrosine phosphorylation, and STAT3 DNA-binding activity in IL-4-induced monocytes. Phosphorylated STAT dimerizes and STAT dimers enter the nucleus. Once in the nucleus, STAT3 activates the transcription of its target genes, including genes for regulation, proliferation, and survival such as cyclin D1, survivin, VEGF, c-myc, Bcl-xL, and Bcl2. Following release from its DNA targets, STAT3 is dephosphorylated in the nucleus and recycles. Inhibition that prevents STAT3 dimerization and nuclear entry or trapping in the cytoplasm of phosphorylated STAT3 efficiently causes cell death [36]. Our observations showing that inhibition of JAK1/JAK2 overcomes M2 gene expression, potentiated by DLL4 to induce apoptosis, which suggests DLL4/Notch1/HES1 signaling interferes with IL-4 signaling at the STAT level. A direct physical interaction between HES proteins and STAT3 has been previously reported and was found involved in glial cell differentiation [37].

Some of the target genes induced by STAT6 can be repressed by Bcl-6. Thus it could also be speculated that repressors such as SOCS1 and Bcl-6 may be involved. The molecular mechanism of the regulatory and pro-apoptotic interplay between DLL4/Notch and IL-4 signaling still needs further investigation [38].

The fact that fully polarized M2 cells are resistant to DLL4-induced apoptosis suggests that the pattern of Notch receptors expressed on M0, M1 and M2 could be a key factor involved in the DLL4 proapoptotic signal. Qualitative and/or quantitative changes in the expression of Notch receptors upon differentiation and polarization may sensitize cells to DLL4 signal. Our data showing several changes in mRNA steady state levels between M0 and M2, including enhanced Notch1 and decreased Notch2 and Notch3 in M2, can support this hypothesis. Interestingly, Notch3 mRNA level was found dramatically increased by DLL4 in the presence of IL-4. The functional consequences of this regulation will need further investigation.

## Conclusion

Our findings further highlight the involvement of cellular interactions and microenvironment in macrophage polarization and plasticity. They indicate a key role for DLL4 in the M1/M2 balance and suggest that elevated DLL4 on activated endothelial cells and M1 macrophages may contribute to an inflammatory amplification loop promoting M1 at the expense of M2 phenotype. Manipulating macrophage subsets to cure cancer is an emerging therapeutic approach [39, 40]. In parallel, the Notch pathway became an attractive therapeutic target and several tools (e.g., γ-secretase inhibitors, neutralizing antibodies against DLL4 or Notch1) that interfere with Notch signaling are currently being developed and tested in various murine cancer models or even in clinical trials [41–43]. Ultimately, our study proposes to combine both approaches by using DLL4 as a new molecular target to control macrophage differentiation.

## Abbreviations

CBF1: C promoter Binding Factor 1
DAPT: N-[N-(3,5-Difluorophenacetyl)-L-alanyl]-Sphenylglycine t-butyl ester
DLL: Delta-like
HES: Hairy enhancer of split
HEY: Hairy related enhancer of split
IRF: Interferon regulatory factor
Jag: Jagged
KLF: Krüppel-like factor
NF-κB: $nuclear factor-κB
NICD: Notch intracellular domain
STAT: Signal transducer and activator of transcription
TLR: Toll like receptor
